# Exploring the Impact of Adaptive Behaviors on Balance: A Comparative Analysis of Static and Dynamic Balance in Athletes With and Without Intellectual Disabilities

**DOI:** 10.1002/brb3.70174

**Published:** 2025-02-18

**Authors:** Elisa Benito‐Martínez, Beatriz Alonso‐Cortés, Mario Fernández‐Gorgojo, Raul Coto Martín, Ricardo Méndez Blanco

**Affiliations:** ^1^ Orden San Juan de Dios Foundation Madrid Spain; ^2^ Health Sciences Department. San Juan de Dios School of Nursing and Physical Therapy Comillas Pontifical University Madrid Spain; ^3^ SALBIS Research Group, Faculty of Health Sciences, Campus of Ponferrada University of León Ponferrada Spain

**Keywords:** adaptive behaviors, static and dynamic balance, intellectual disability

## Abstract

**Background:**

Individuals with intellectual disability (ID) shows less development on their motor skills. Understanding the interaction between intellectual and physical performance will provide valuable information for the design of personalized interventions and support programs able to enhance active participation of these individuals.

**Method:**

A descriptive‐analytical study with 93 participants (59 with ID) was designed to evaluate both static and dynamic balance in athletes. In addition, the relationship between balance and adaptive behaviors was analyzed to determine whether static and dynamic balance is affected to a greater or lesser extent depending on the level of adaptive skills or intellectual capacity.

**Results:**

Significant differences were found in balance's variables between individuals with and without ID. IQ measurements (processing speed) and adaptive behaviors showed a correlation with some balance variables. In athletes with less ID levels, the correlation between balance and adaptive behaviors is more notable.

**Conclusions:**

There are clear differences in both static and dynamic balance between individuals with and without intellectual disabilities. In addition, the level of adaptive skills and processing speed is related to balance, with those having lower levels of adaptive skills experiencing greater difficulties in maintaining better balance

## Graphical abstract

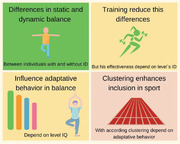

## 1 Introduction

Intellectual disability (ID) is characterized by intellectual performance below average and significant limitations in two or more adaptive behaviors. Adaptive skills encompass several areas and are commonly classified into three main domains: conceptual skills (such as communication and self‐care), social skills (interpersonal and responsibility), and practical skills (living and work daily activities) (Schalock, Luckasson, and Tassé 2021b). Assessing adaptive behaviors is essential for understanding individual needs and designing personalized interventions to promote independence and active participation in society (Giummarra, Randjelovic, and O'Brien 2022). Adaptive behavior is understood as “the collection of conceptual, social, and practical skills that people have learned to function in their daily lives” (Luckasson et al. 2002). Limitations in adaptive behavior affect both daily life and the ability to respond to life changes and environmental demands. Previous investigations confirm that the development of these skills can widely vary among individuals with ID (Mattie et al. 2023). The adaptive behaviors' system assessment (ABAS II test) has been used in previous investigations to perform the assessment of adaptive behaviors (De la Torre et al. 2016; Doane and Salekin 2009). The diagnosis of ID requires an Intelligence Quotient (IQ) test (Jouira et al. 2020), which must be conducted before the age of 22, according to the American Association on Intellectual and Developmental Disabilities (AAIDD)(Schalock, Luckasson, and Tassé 2021a).

On the other hand, locomotor system dysfunction and limited functional capacity are also other characteristics commonly associated with ID. Research on individuals with ID has found that they perform less effectively on motor skill tests in comparison to those who are cognitively and intellectually healthy (Bahiraei et al. 2018). These functional limitations can lead to a negative emotional impact and could contribute to further deterioration of their physical and mental development, as well as impede their integration among individuals without ID (Mikolajczyk and Jankowicz‐Szymanska 2015).

Currently, a significant amount of research is underway to develop different therapeutic strategies for improving balance in individuals with ID (Kaya et al. 2023; Maïano, Hue, Lepage, et al. 2019; Maïano, Hue, Morin, et al. 2019). These individuals form a cohort characterized by balance challenges arising from a lack of coordination among the visual, vestibular, and proprioceptive systems (Blomqvist et al. 2013; Enkelaar et al. 2012). It has been observed that people with ID have weaker balance control and tend to have a higher degree of swaying compared to those without intellectual disorders (Rigoldi et al. 2011), increasing the risk of falling (Ho et al. 2019; Smulders et al. 2013). When the increment of the risk of falling occurs in older individuals, it can lead to injuries, institutionalization, and have a negative effect on the quality of life (Finlayson et al. 2010; Frighi et al. 2022).

However, previous research suggests that a decrease in central cognitive processing is the primary cause of balance impairment (Marsh and Geel 2000). In addition, due to incomplete development and premature aging, adults with ID have a balance deficit similar to healthy children (Enkelaar et al. 2012). This balance impairment is exacerbated when performing simultaneous cognitive tasks, supporting the hypothesis that the neurocognitive systems plays a determining role in such deficits (Van Biesen et al. 2018).

Since balance is essential for daily movement, activities, and sports practice, healthcare professionals should be aware of the importance of its monitorization (Cuesta‐Vargas and Giné‐Garriga 2014; Dellavia et al. 2009). The assessment of the postural control system performance is generally conducted through static and/or dynamic testing using stabilometric or balance platforms or several clinical tests (Blomqvist et al. 2012). Despite the objectivity and accuracy of stabilometric platforms data, clinical tests are frequently used as balance assessment tools in individuals with ID (Bahiraei, Hosseini, and Lou 2023; Blomqvist et al. 2012; Maïano, Hue, Morin, et al. 2019).

The most common tests used to measure balance are the Single Leg Stance (SLS) test with or without open eyes (Cuesta‐Vargas and Giné‐Garriga 2014), the Time Stand Test (Birmingham 2000), the Functional Reach Test (FRT) (Duncan et al. 1990), and the Expanded Timed Get Up and Go (ETGUG) Test (Salarian et al. 2010).

Cuesta‐Vargas et al. carried out in 2014 a comparative research to find out what tests should be chosen when assessing balance (Cuesta‐Vargas and Giné‐Garriga 2014). After using a principal component analysis to these tests, the results indicated that the FRT and the SLS had greater relevance in assessing static balance, while the ETGUG and Time Stand Test were primarily associated with dynamic balance assessment.

Research on balance in young athletes or active individuals with ID has been extensively documented, covering comparisons between athletes and nonathletes or sedentary individuals with ID (Blomqvist et al. 2013; Jouira et al. 2021), as well as between athletes with and without ID (Blomqvist et al. 2017; Dellavia et al. 2009; Jouira et al. 2021; Van Biesen et al. 2018). However, the results obtained in these studies were inconclusive. In addition, there is no strong evidence supporting balance improvement through specific intervention programs for active young individuals with ID (Maïano, Hue, Morin, et al. 2019). Cuesta‐Vargas et al. defend in different studies how improving physical outcomes can prevent falls by enhancing the balance of individuals with ID, even though the variability in results could be linked to the type and duration of the tests performed, as well as the intrinsic characteristics of the sample (Cuesta‐Vargas and Giné‐Garriga 2014).

Another interesting issue about balance in young athletes with ID to be considered has to do with the influence that intellectual performance and adaptive behaviors may have. Understanding the interaction between these variables can not only provide valuable information but also establish the groundwork for the design of personalized interventions and support programs able to enhance both the quality of life and active participation of these individuals. Yet, there has not been sufficient research conducted to address the correlation between these variables in young athletes with ID. Therefore, the main aim of this research was to evaluate both static and dynamic balance in young athletes with ID and seek to compare balance test results with young athletes without ID (control group). In addition, the relationship between balance and adaptive behavior performance was analyzed to determine whether static and dynamic balance is affected to a greater or lesser extent depending on the level of adaptive skills or intellectual capacity.

## 2 Methods

A descriptive‐analytical study was designed to achieve the posed objectives. Evaluations were carried out during the months of April to July 2022. The Bioethics Committee of Carlos III Clinic Hospital approved this research before the start of the study (22/106). The research was carried out following the Declaration of Helsinki of 1975.

### 2.1 Sample

A total of 93 participants took part in the research. Inclusion criteria required all participants to engage in at least 4 h of sports per week. In addition, individuals in the ID group had to provide legal documentation of at least 33% disability issued by the Spanish government, with the etiology of this disability being intellectual in nature. Exclusion criteria included suffering from any type of injury or illness that could reduce or impair balance at the time of the study, such as ear diseases. Furthermore, in subjects with ID, exclusion criteria included not showing sufficient interest in performing the psychological tests, taking medication that could limit the results in the psychological tests to be performed, or having severe speech or communication difficulties as defined in the ABAS II and Wechsler Adult Intelligence Scale (WAIS‐IV tests). The 59 subjects with ID (73.38 ± 18.63 kg, 1.67 ± 0.08 m, and 25.32 ± 4.34 years) assessed were linked to three foundations for people with ID of Palma de Mallorca city in Spain, and they were recruited through the *Federació d'Esports Adaptats de les Illes Balears*. All participant's legal guardians were informed by ID foundation's coaches at a previous session, and informed consent was signed in each case to allow participation in the study. The 34 subjects without ID (68.36 ± 9.59 kg, 1.69 ± 0.07 m, and 21.03 ± 1.04 years) assessed were students of Pontificia Comillas's University. All of them were informed of the procedure at a previous session and also had to sign the corresponding informed consent to participate in the study as well.

### 2.2 Testing Procedures

The initial sociodemographic data collection for both groups included date of birth, gender, primary sport practiced, and leg dominance (it was assessed with the Harris Test of Lateral Dominance; Ochoa Maigua &Tipanluisa Tandalla 2023), participants with ID were able to respond themselves in all cases. In addition, balance data were collected through the stabilometric platform. In addition, participants with ID completed test measuring IQ (WAIS IV), test measuring adaptive behaviors (ABAS II), and the Functional Reach Test, as well as the Timed Up and Go Test.

All participants underwent an initial training session for the tests. If any subject had doubts before the beginning of the tests, a visual demonstration of the test was provided again by the research team.

## 3 WAIS IV and ABAS II Test

In relation to the WAIS IV testing procedure, only scores from the perceptual reasoning and processing speed scales were considered. This decision was informed by the research team's considerable expertise, as well as by previous studies (which are still unpublished) demonstrating that these scales exhibit the strongest correlation with sports performance. Concerning the ABAS II, the inquiries were spread across 10 domains and categorized into three subscales (conceptual, social, and practical skills). These were responded to by the athlete with ID, in collaboration with their caregiver, who needed to be a closely associated individual residing with them (Luckasson et al. 2002). Finally, the test provides a global score in addition to scores for the three subscales. Both assessments were conducted by a psychologist specializing in people with ID.

### 3.1 Stabilometry Platform

Static balance measurements were evaluated utilizing the Zebris FDM‐S Plate‐Medical GmbH, with dimensions of 1370 mm × 535 mm × 15 mm (*l* × *b* × *h*). The size of the measurement area is 1220 mm × 474 mm (*l* × *b*). The procedure involved instructing the participants to stand barefoot on the platform, maintaining a straight‐ahead gaze for a duration of 30 s. Subsequently, the same data were collected, this time for 20 s with closed eyes. During this test, a participant with arms placed freely along their body sides stands on both lower limbs, which are distanced from each other at the pelvis width. A designated individual stood behind the athlete throughout to prevent any potential loss of balance. Subsequently, the athlete was instructed to stand on one leg, and data were gathered following the same procedure for both sides. In cases where participants were unable to complete the monopodal acquisition, it was utilized as an outcome measure.

The repeatability of foot positioning on the Zebris PDM‐L platform was conditioned by the lines permanently adhesive on the platform. The center line was marked and coincided during subsequent static tests with the center line of the body of the studied person. In all tests, subjects were instructed to keep their feet within the platform, and the center of the foot was made to coincide with the line that horizontally divided the platform in half. The feet were placed at hip height with the same distance from the midline, but a specific location or distance from the midline was not defined for their placement since this depended on the size of the subject. No visual feedback was provided.

Data were collected on the lateral deviation of the center of pressure in the anteroposterior and lateral planes, the confidence area, and the displacement speed of the center of pressure.

### 3.2 Time Stand Test

A chair without armrests was placed, and the athlete was asked to sit on it. The athlete was instructed to keep their arms crossed over their chest and their feet flat on the ground, separated at shoulder width. The time taken by the athlete to stand up and sit back down 10 times was recorded. In the standing position, the athlete assumed a fully upright position with extended knees.

### 3.3 Functional Reach Test

A 1‐m‐long line was placed on the wall at approximately shoulder height of the athlete, who stood sideways against the wall with their arm extended and their shoulder at a 90° angle of flexion so that it was supported against the wall and their fingertips extended just at the beginning of the drawn line. Without lifting the arm from the wall, the athlete had to advance their hand along the line as far as possible while being able to return to the starting position. A rotation of the trunk towards the opposite side of the wall was allowed, and the gained length was recorded (Cuesta‐Vargas and Giné‐Garriga 2014; Duncan et al. 1990).

### 3.4 Expanded Timed Get Up and Go Test

A chair without armrests was placed at the starting point, and a line was drawn on the ground 10 m away from the front legs of the chair. The athlete stood up from the chair from a seated position with their feet flat on the ground, separated at shoulder width, and with their arms in a free position but not allowed to be used to stand up. The athlete walked as quickly as possible without running to the line located 10 m away, crossed it, turned right or left, and returned to the chair to assume the seated position. The total time of the exercise was recorded, as well as the partial time from 2 m before crossing the line to 2 m after crossing it again (Salarian et al. 2010).

#### 3.4.1 Data Analysis

A descriptive analysis was conducted on the study variables, utilizing the median and range.

Subsequently, the samples were compared based on the presence of ID. Nonparametric tests (Mann–Whitney *U* test) were employed, and the effect size was evaluated using Hedges' *g*. Finally the correlation between intellectual variables and stabilometric variables was examined, employing Spearman's test in all instances.

Statistical analysis was carried out using IBM SPSS Statistics version 28.0.1.1 software. The threshold for statistical significance was set at *p* < 0.05.

## 4 Results

The descriptive data of the tests conducted on the stabilometric platform, as well as the results of the Mann–Whitney test and the effect size assessed according to Hedges' *g*, are presented in Table 1.

**TABLE 1:** Descriptive data and Mann–Whitney *U* test results for static and dynamic balance variables. The effect size was assessed according to Hedges' *g*.

		Without ID (*n* = 34)	With ID (59)	Mann–Whitney *U*		
		Median (range)	Median (range)		*p*	Hedges' *g*
Bipodal	Lateral deviation	2.90 (7.40)	28.70 (89.50)	148.5	0.000	1.452
Open eyes	Anteroposterior deviation	7.00 (11.70)	35.80 (95.10)	172.50	0.000	1.553
	Confidence area	14.80 (71.10)	75.30 (1698.90)	154.00	0.000	0.594
	Speed	5.20 (10.60)	7.20 (35.50)	544.00	0.006	0.609
Bipodal	Lateral deviation	4.50 (8.40)	25.40 (89.80)	148.00	0.000	1.347
Close eyes	Anteroposterior deviation	9.40 (10.80)	30.30 (78.80)	203.50	0.000	1.439
	Confidence area	32.30 (112.7)	82.00 (1108.90)	354.00	0.000	0.653
	Speed	8.70 (10.20)	11.00 (382.40)	668.50	0.097	0.198
Unilateral	Lateral deviation	10.80 (22.80)	100.70 (223.40)	143.00	0.000	1.850
Dominant	Anteroposterior deviation	17.20 (40.40)	26.80 (83.70)	822.50	0.015	0.645
Open eyes	Confidence area	112.50 (353.10)	367.90 (8469.40)	323.00	0.000	0.700
	Speed	22.90 (35.60)	43.10 (245.90)	365.00	0.000	0.900
Unilateral	Lateral deviation	10.00 (8.00)	41.00 (191.00)	208.50	0.000	1.270
No Dominant	Anteroposterior deviation	15.60 (19.00)	32.80 (100.00)	367.00	0.000	1.000
Open eyes	Confidence area	119.80 (191.30)	448.60 (10305.90)	228.00	0.000	0.670
	Speed	20.00 (29.30)	42.70 (241.90)	224.50	0.000	0.920
Unilateral	Lateral deviation	21.10 (45.30)	91.80 (222.10)	108.50	0.000	1.760
Dominant	Anteroposterior deviation	29.00 (55.60)	27.70 (89.20)	759.00	0.392	0.07
Close eyes	Confidence area	476.90 (3299.60)	1401.50 (9788.10)	349.00	0.000	0.900
	Speed	56.30 (83.30)	85.00 (228.40)	466.00	0.001	0.810
Unilateral	Lateral deviation	21.60 (29.10)	58.60 (183.00)	378.50	0.000	1.040
No dominant	Anteroposterior deviation	32.80 (77.70)	33.40 (93.40)	791.00	0.567	0.05
Close eyes	Confidence area	597.30 (3001.50)	1145.50 (12708.20)	524.00	0.003	0.680
	Speed	58.00 (185.30)	70.40 (229.30)	640.00	0.056	0.460
Time Stand Test			22.10 (34.00)			
Functional Reach Test			31.50 (35.00)			
Stand and go global test			13.29 (21.00)			
Stand and go gire test			3.18 (5.00)			

Significant differences were found in all measured variables utilizing the stabilometric platform between individuals with and without ID, excluding the speed of movement in bipedal support with eyes closed and in unipedal support with eyes closed on the nondominant leg, as well as unilateral anterior–posterior deviations with eyes closed.

Regarding the correlation analysis between IQ measurements and balance variables, significant correlations were observed solely between the area of confidence in bipedal support—both with eyes closed and eyes open—and the processing speed subscale of the WAIS IV. In addition, correlations were found between the closed eyes test with the dominant leg and the processing speed subscale of the WAIS IV. No correlation was identified between the data obtained from the stabilometric platform and the perceptual reasoning category of the WAIS IV (Table 2).

**TABLE 2:** Spearman correlation between the processing speed variable (WAIS IV) and balance variables.

	WAIS IV processing speed							
	Open eyes				Close eyes			
	Lateral deviation	Anteroposterior deviation	Confidence area	Speed	Lateral deviation	Anteroposterior deviation	Confidence area	Speed
Bilateral								
Spearman's rho								
Correlation coefficient	0.073	0.065	−0.480^**^	−0.380^*^	0.168	−0.009	−0.390^*^	−0.233
Significant (bilateral)	0.697	0.727	0.006	0.035	0.367	0.963	0.03	0.207
Unilateral dominant								
Spearman's rho								
Correlation coefficient	0.268	0.038	−0.288	−0.103	−0.189	0.167	−0.464^**^	−0.171
Significant (bilateral)	0.145	0.84	0.116	0.582	0.308	0.37	0.009	0.357
Unilateral no dominant								
Spearman's rho								
Correlation coefficient	0.304	0.041	−0.1	0.058	0.052	−0.117	−0.202	−0.124
Significant (bilateral)	0.096	0.828	0.593	0.758	0.783	0.531	0.276	0.507
*N*	31	31	31	31	31	31	31	31

*p < 0.05 and **p < 0.01.

Regarding the adaptive behaviors variables, they showed a correlation with the area of confidence in unipedal support, both with eyes closed and open when the supporting leg is dominant, and only with open eyes on the nondominant leg. These correlations were observed in both the overall scores of the ABAS II and in the subcategory of conceptual adaptive behaviors (Table 3).

**TABLE 3:** Spearman correlation between the variables of the ABAS II test and the confidence area. ABAS II CAG (global adaptive behavior).

	Bilateral		Unilateral dominant		Unilateral no dominant	
	Confidence area		Confidence area		Confidence area	
	Open eyes	Close eyes	Open eyes	Close eyes	Open eyes	Close eyes
ABAS II CAG						
*R*	−0.096	0.037	−0.356^*^	−0.439^**^	−0.333^*^	−0.255
*p*	0.55	0.816	0.022	0.004	0.033	0.108
ABAS II conceptual						
*R*	−0.119	0.02	−0.385^*^	−0.435^**^	−0.382^*^	−0.245
*p*	0.459	0.899	0.013	0.005	0.014	0.122
ABAS II social						
*R*	−0.310^*^	−0.142	−0.284	−0.264	−0.217	−0.153
*p*	0.049	0.376	0.072	0.095	0.174	0.338
ABAS II practical						
*R*	−0.014	0.087	−0.277	−0.354^*^	−0.241	−0.168
*p*	0.933	0.588	0.08	0.023	0.129	0.295

*p < 0.05 and **p < 0.01.

The outcomes of the correlation analysis between adaptive behavior levels and static as well as dynamic balance variables revealed no statistically significant distinctions. However, among athletes with IQ levels three standard deviations below the mean, the correlation between the Time Stand Test and adaptive behaviors starts to become more notable (specifically, with respect to general, conceptual, and practical skills, Spearman's *R* = 0.448, *p* = 0.032; *R* = 0.585, *p* = 0.030; and *R* = 0.539, *p* = 0.080, respectively)

## 5 Discussion

The descriptive statistics of the sample revealed significant heterogeneity among subjects, stemming from the wide range of diagnoses and proportions of ID present. Regarding disparities between individuals with and without disabilities, the majority of measured stabilometric variables exhibit notable differences. Notably, certain variables, such as lateral displacement of the center of gravity in bipedal and unipedal support, are up to 10 times higher in the ID population (10.80 vs. 100.70 and 2.90 vs. 28.80, respectively). Lee, Lee, and Song (2016) concluded, however, that although intellectual capacity influences concentration and action execution, it does not significantly correlate with the ability to maintain balance. This statement aligns with the findings of Laatar et al. (2023), who confirmed that dual‐task conditions (recognition memory test and balance test) affect the postural performance of both people with and without ID. However, other studies, such as those conducted by Dellavia et al. (2009), have shown that balance or equilibrium is impaired in people with ID. Franciosi even determined that motor coordination or dynamic balance measured by the Timed Up and Go Test was a predictor of the performance of athletes with moderate ID in the 60‐m race (Franciosi et al. 2010).

In addition, Dehghani and Gunay (2015) demonstrated how training programs could improve both static and dynamic balance in 10‐year‐old children with ID. Similarly, Mikołajczyk and Jankowicz‐Szymańska (2014) showed improvement in static balance with open and closed eyes with an exercise program, as well as Jouira (Jouira et al. 2022, Jouira et al. 2024) confirmed that participation in running training is linked to improved dynamic balance, while neuromuscular training has the potential to enhance both static and dynamic balance. Hence, we can infer that given our ID population's involvement in sports and their training in balance, the disparities observed with the non‐ID population under study are less pronounced than anticipated, especially when compared to ID populations not engaged in any physical activity.

Blomqvist's and Carmeli's (Blomqvist et al. 2018; Carmeli et al. 2005) studies concluded that postural balance in individuals with ID, both young and adult, is not more reliant on vision compared to individuals without ID. Similarly, Mikołajczyk and Jankowicz‐Szymańska (2014) state that people with disabilities have greater concentration when performing tasks with closed eyes. However, other authors argue that the lack of visual input can increase the range of movements of the center of mass and affect static postural balance in ID athletes, more noticeably than in non‐ID population, thus, emphasizing the importance of visual input in this aspect (Jouira et al. 2021). This aligns with the findings of our study involving athletes with ID. In tests conducted with closed eyes, the majority were unable to achieve the minimum required time in unipedal support, necessitating support from the contralateral foot to prevent falling. This limitation constitutes the primary reason why the results of our stabilometric tests with closed eyes lack decisiveness.

While athletes with ID may experience challenges in utilizing proprioceptive signals in the absence of visual input, targeted sports practice and strength and balance training have been shown to enhance the effectiveness of the proprioceptive system. This fosters the development of the capacity to swiftly transition between sensory systems, leading to improved performance in postural balance (Paillard 2017).

Regular physical activity, strength and balance training programs, and specific sports practices appear to be key elements in improving postural balance in people with ID (Lee, Lee, and Song 2016).

The big question is to what extent balance training in people with ID can improve this capacity and if the level of intellectual ability is related to this capacity.

Based on this, our study demonstrates that regarding processing speed, there is a moderate correlation with confidence area in bipedal support (−0.48, *p* = 0.006). In addition, if the sample is segmented, in those athletes who present a lower level of IQ (processing speed < 53), correlations appear with the confidence area and the speed of movement. These correlations are not present in athletes who have a higher IQ, closer to the population average. It could, therefore, be thought that with a higher level of disability, static balance may be more affected. This statement is consistent with the data obtained in the study by Blomqvist et al. (2013), where they evaluated the speed of displacement of the center of gravity in bipedal and unipedal support of people with and without ID, finding that the speed of displacement in all tests was higher in the group with ID. This aligns with Cuesta‐Vargas and Pérez‐Cruzado (2014) but disagrees with Van Biesen et al. (2018), which confirms that IQ is not related to physical abilities. This variation could stem from the fact that the study carried out by Van Biesen exclusively involved athletes participating in international championships (VIRTUS), where access is limited to athletes with higher intellectual abilities without considering varying levels of disability.

Regarding adaptive behaviors variables, the conceptual skills area correlates with the confidence area in unipedal support, both with closed and open eyes when the supporting leg is dominant and only with open eyes on the nondominant leg. Perhaps this could be due to the inability of most athletes to complete the 20‐s unipedal support test with closed eyes on the nondominant leg as they lost balance before finishing, thus, biasing the data. At the bipedal support level, only social skills present a significant correlation in the test with open eyes.

The increase in postural oscillation in these individuals leads to dysfunction in static balance, generating subsequent disorders in the dynamic balance necessary to perform physical functions. This implies a lack of ability to integrate visual, proprioceptive, and balance information, essential elements to maintain a balanced posture and perform appropriate coordinated movements.

## 6 Conclusions

Differences in static and dynamic balance were observed between individuals with and without IDs. Training has the potential to reduce disparities in static and dynamic balance between populations with and without IDs. The effectiveness of training may be influenced by the levels of processing speed and adaptive behaviors.

The level of adaptive skills and processing speed is related to balance, with those having lower levels of adaptive skills experiencing greater difficulties in maintaining better balance.

Additional investigation is needed to delve into the connection between variables defining ID and static and dynamic balance, as well as to examine how training can improve these abilities.

Furthermore, one of the main limitations of this study could be the significant heterogeneity of ID diagnoses within the sample. It is advisable to explore methods to account for these diagnoses in order to segment the sample effectively.

## Author Contributions

**Elisa Benito‐Martínez**: conceptualization, investigation, writing–original draft, writing–review and editing, methodology, formal analysis, project administration, supervision, resources. **B. Alonso‐Cortés**: methodology, data curation. **M. Fernández‐Gorgojo**: writing–original draft, writing–review and editing. **R. Coto Martín**: validation, writing–review and editing, formal analysis. **R. Méndez Blanco**: conceptualization, investigation, writing–original draft, data curation, resources.

## Ethics Statement

All study participants received an informative document about the study content and procedures, and the corresponding informed consent was either signed by the athlete himself or by his legal custodian, if necessary. The approval of the Bioethics Committee of Carlos III Clinic Hospital was obtained with number (22/106).

## Conflicts of Interest

The authors declare no conflicts of interest.

### Peer Review

The peer review history for this article is available at https://publons.com/publon/10.1002/brb3.70174.

## Data Availability

The data that support the findings of this study are available from the corresponding author upon reasonable request.
